# Precision Ice Detection on Power Transmission Lines: A Novel Approach with Multi-Scale Retinex and Advanced Morphological Edge Detection Monitoring

**DOI:** 10.3390/jimaging10110287

**Published:** 2024-11-08

**Authors:** Nalini Rizkyta Nusantika, Jin Xiao, Xiaoguang Hu

**Affiliations:** State Key Laboratory of Virtual Reality Technology and Systems, School of Automation Science and Electrical Engineering, Beihang University, Beijing 100191, China; nusantikanalini@buaa.edu.cn (N.R.N.); xiaoguang@buaa.edu.cn (X.H.)

**Keywords:** power transmission line icing, ice detection, image segmentation, edge detection, multi-scale Retinex, morphological operations, ice thickness measurement, binocular cameras, monitoring systems

## Abstract

Line icings on the power transmission lines are dangerous risks that may lead to situations like structural damage or power outages. The current techniques used for identifying ice have certain drawbacks, particularly when used in complex environments. This paper aims to detect lines on the top and bottom in PTLI with low illumination and complex backgrounds. The proposed method integrates multistage image processing techniques, including image enhancement, filtering, thresholding, object isolation, edge detection, and line identification. A binocular camera is used to capture images of PTLI. The effectiveness of the method is evaluated through a series of metrics, including accuracy, sensitivity, specificity, and precision, and compared with existing methods. It is observed that the proposed method significantly outperforms the existing methods of ice detection and thickness measurement. This paper uses average accuracy of detection and isolation of ice formations under various conditions at a percentage of 98.35, sensitivity at 91.63%, specificity at 99.42%, and precision of 96.03%. Furthermore, the accuracy of the ice thickness based on the thickness measurements is shown with a much smaller RMSE of 1.20 mm, MAE of 1.10 mm, and R-squared of 0.95. The proposed scheme for ice detection provides a more accurate and reliable method for monitoring ice formation on power transmission lines.

## 1. Introduction

Ice buildup on power transmission girds can pose a significant risk to the electric power system. These conditions can result in structure collapse, power outages in some of the provinces, and localized economic losses. Power transmission lines can sag, break, or even collapse under the extra burden of the ice load, which in turn affects supply to some of the regions as well as poses some risks [1,2,3,4,5,6]. The design of systems to detect and prevent ice buildup on transmission lines is essential to maintain the integrity of infrastructure and minimize the associated economic and safety risks [7,8]. Transmission line structure in areas such as mountains or regions that have extremely cold climates exposes cold water droplets to the lines. For instance, within a few hours water droplets form and start freezing into ice layers [9,10,11,12,13,14]. These layers will adhere to conductors, insulators, and supporting structures, leading to network breakdowns, tower collapses, and other accidents, which have a great impact on the operation of the transmission line and seriously threaten the reliability and safety of power system operation [15], bring substantial economic losses to enterprises [16,17], and bring security risks to the country and society [13,18]. Between 1980 and 2000, several cities in the United States, the United Kingdom, Canada, Russia, Norway, and other countries experienced transmission network failures caused by snowstorms [12,19]. Several countries have experienced significant ice load disasters on power transmission lines, resulting in widespread impacts of the problem [20,21,22]. In late January 1998, a North American ice storm struck the central and eastern United States and then the northeastern United States; millions of people experienced power outages due to downed power lines, with electrical damage totaling over USD 5 billion. In the same year (1998), an ice storm struck Ontario and Quebec, particularly disrupting power supplies and infrastructure and causing losses amounting to approximately USD 3 billion. Siberian ice storms that occurred in 2001, as well as others in 2002, have exposed Russia’s power transmission circuits to severe ice loads which have impacted transmission infrastructures extensively and also affected Russia’s economy. An ice event on the transmission lines in northern France in the year 2005 led to problems with power line structures and a blackout that affected hundreds of thousands of people and required extensive repair costs. In 2005 and 2008, southern China experienced the most serious ice disaster in history [12]. Among the damages, the loss of Yunnan’s power grid was the most serious, with direct economic losses of more than CNY 10 billion [23,24]. Ice disasters disrupted most of the power line infrastructure, which affected millions of people and cost roughly CNY 56 billion (USD 8 billion). In February 2021, an ice-laden power transmission disaster in Texas left millions of homes without power in the cold weather, exposing infrastructure weaknesses and causing billions of dollars in economic losses [25].

Ice accumulation on transmission lines poses a real threat to the stability of the power transmission network, and, therefore, regular detection of ice on transmission lines becomes essential. However, this detection is easily hampered by climatic features such as variations in visibility and very low image contrast, which poses problems with image processing-based detection techniques that can lead to false alarms or complete failure of detection [26,27]. Several methods have been applied to determine the monitoring of ice on power transmission lines, namely inspection by human eyes, vehicles, meteorological models, mechanical models, and the use of sensors. Manual inspection is time-consuming, expensive, and is always considered impractical, especially in hilly areas. Vehicle monitoring is accurate in inspection but is limited to the surrounding areas or urban environments only. Meteorological and mechanical models provide more information on the subject but are detailed and may need to be more reliable due to meteorological disturbances. Electromagnetic disturbances are one of the major problems faced by sensor-based methods, which, apart from providing mass and thickness data, also have difficulties when applied in various conditions [26,28]. In recent years, power transmission line icing (PTLI) has developed into an indicator that is monitored by image processing, which is based on the enhancement of computer vision [29]. By using cameras on the PTLI tower or using UAVs equipped with cameras on board, operators can efficiently monitor and analyze ice formation on transmission lines. As mobile and safe platforms, UAVs improve the capabilities and performance of PTLI through the acquisition and analysis of images to provide improved edge and contour detection for better ice thickness measurements [30,31].

Ice detection on transmission lines is an important basic part of PTLI monitoring. The goal is to identify the upper and lower boundaries of the closed transmission line because it directly affects the thickness measurement results. Generally, the detection stage is on the transmission line consisting of two parts: edge detection and line detection. Traditional methods for detecting ice on power transmission lines use Canny edge detectors and Hough transforms for line detection. However, these methods face significant challenges, including sensitivity to interference, difficulty in complex backgrounds, problems with background interference, and poor lighting [32,33]. In previous methods, improved Canny edge detection using hybrid filters and Hough transforms for line detection were applied to PTLI monitoring. However, there are still noise and under-detected lighting conditions [34]. Another method for monitoring ice on transmission lines using a combination of multi-scale Retinex image enhancement, multi-threshold segmentation, ROI, and morphology for edge detection and connected components with component labeling for line detection has been carried out. In this scheme design, it successfully segmented PTLI and the results also outperformed the traditional Canny and Hough transform methods. However, there are PTLI regions that are not segmented, so improvements are needed [10]. Liang S. et al. proposed a linear detection algorithm based on the LSD algorithm to detect ice thickness on transmission lines. First, image processing methods such as image preprocessing, morphological processing, and edge detection are used to preprocess the ice image on the transmission line. Then, the edge of the ice image is detected using the LSD algorithm. However, this algorithm has some limitations. For example, in most cases, light, fog, and snowy conditions can hinder the image quality, hence lowering the level of detection. This algorithm optimized for straight lines may have difficulty with irregular ice shapes, and its effectiveness depends heavily on accurate image preprocessing [35]. Weng B. et al. proposed a novel method combining machine vision and machine learning algorithms to identify the thickness of high-voltage transmission lines. The improved Canny edge detection algorithm, Hough transform, improved K-means clustering, and least squares fitting were adopted alternately to find the conductor edge. This method is accurate and reliable; however, the complexity of the method requires significant computational resources and may be less suitable for real-time analysis. In addition, the performance of this algorithm may be sensitive to the tuning parameters, which require continuous adjustment based on specific conditions [28]. Li W. et al. developed a lightweight convolutional neural network, WearNet, to realize automatic scratch detection for components in sliding contact, such as those in metal forming [36]. However, WearNet showed results for surface scratch detection in metal forming, and its application to ice detection on power transmission lines with image processing will face challenges related to domain-specific feature extraction, real-time processing complexity, and data generalization.

This research aims to overcome the limitations of current methods and improve the accuracy and reliability of PTLI identification by introducing several contributions:

- Multistage integration for detecting ice formation on power transmission lines: this scheme integrates advanced image processing techniques into a multistage pipeline specifically designed for ice detection on power transmission lines.
- Dynamic context and parameter adaptation: this scheme introduces high adaptability through dynamic parameter adjustment based on contextual image characteristics.
- Multi-scale enhancement and perceptual color preservation to distinguish ice from power lines: adaptive Retinex and perceptual color preservation are used to enhance the visibility and sharpness of power lines and ice formations.
- Combination of edge detection techniques and enhancement with adaptive blending: The combination of multiple edge detection methods and adaptive thresholding: this scheme generates a comprehensive edge map for PTLI.
- Interactive visualization for real-time monitoring: the scheme also provides an enhanced interactive dynamic visualization feature that enables monitoring in real-time.
- Graph-based segmentation with geometric constraints for higher accuracy: the segmentation stage combines graph construction with geometric constraints specifically designed to separate ice from power lines.
- Multi-resolution processing to ensure accuracy in every detail: By using multi-resolution processing at nearly every stage, the scheme enables detailed analysis at multiple scales. This method ensures that both fine details and coarse structures of the ice can be identified well, which is especially useful in detecting more complex ice formations.

In general, this research scheme combines multistage and modifies or improves the methodologies to meet the specific challenges in PTLI. This contribution presents that research scheme presents a new method that not only implements but also develops techniques for more accurate, adaptive, and real-time monitoring, making it an innovative and relevant solution in the field of power infrastructure maintenance.

## 2. Materials and Methods

### 2.1. Materials

#### 2.1.1. Imaging Equipment

**Figure 1:**
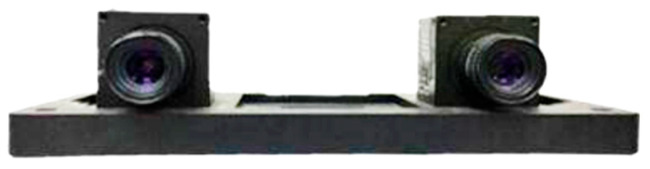
Daheng binocular vision camera structure.

This paper utilizes high-resolution binocular cameras, specifically two DH-HV1351UM-ML cameras from Daheng Imaging Company (based in Beijing, China), to capture images of ice on power transmission lines. The cameras, spaced 8 cm apart, offer a resolution of 1280 × 1024 pixels, 10-bit analog-to-digital conversion, and a frame rate of 15 fps. These cameras are designed for precision, featuring adjustable digital gain, flexible shutter times, and programmable control for image settings. The camera size and low power consumption make it suitable for integration into an unmanned aerial vehicle (UAV) or other platforms for efficient PTLI monitoring. The physical image is shown in Figure 1.

#### 2.1.2. Software Tools

The ice image processing algorithm on the power transmission network includes Python 3.11 functions, OpenCV 4.0, and several other algorithms developed internally. The importance of image processing in this study can be performed by using functions such as OpenCV for edge detection, segmentation, and object recognition, which are carried out through Python functions. Preprocessing and special tasks such as increasing image contrast and removing noise are completed by special algorithms, which are implemented with Python functions. This study also provides a fast and efficient image-processing method with Python and OpenCV programming languages, both of which are supported by additional algorithm inputs developed separately for the system.

#### 2.1.3. Data Set

**Figure 2:**
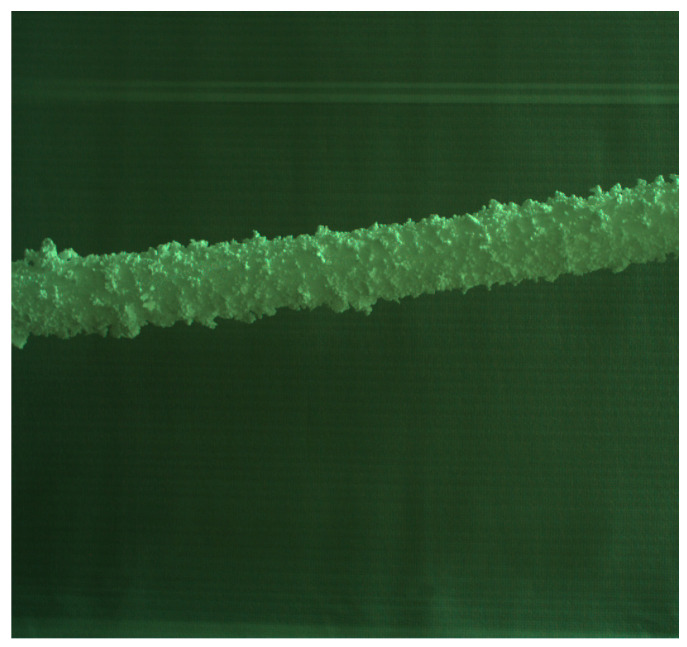
Simulation of ice formation on transmission lines.

In order to facilitate the collection of ice-covered PTLI image data, a series of scenes were independently constructed to simulate the ice-covered transmission line. This study used long cylindrical pearl cotton (Expandable Polyethylene, EPE) to simulate the transmission channel. The EPE utilized in this study was sourced from Guangdong Chuanling New Material Technology Co., Ltd., located in Guangdong, China. Then, polystyrene foam (Expanded Polystyrene, EPS) was attached to the surface to simulate ice cover. Expanded Polystyrene foam (EPS) is attached to the surface to simulate an ice load layer. The EPS used in this study was sourced from Hebei Sashang Technology Co., Ltd., located in Shijiazhuang City, Hebei Province, China. The PTLI image that was created is shown in Figure 2.

The dataset consists of images taken under various conditions to represent possible scenarios comprehensively. The dataset in this study consists of 200 PTLI images taken at different times with varying lighting and background conditions. PTLI images were taken using a binocular camera under various indoor conditions, and 200 images were taken randomly for the experimental study of this research. The images are of artificial ice on the transmission line and white sky. The proposed method will be tested on different backgrounds and with various lighting conditions to ensure the efficiency of the system. This method covers all aspects and thus enables the detection of ice formation on power transmission lines as accurately as possible.

### 2.2. Methods

**Figure 3:**
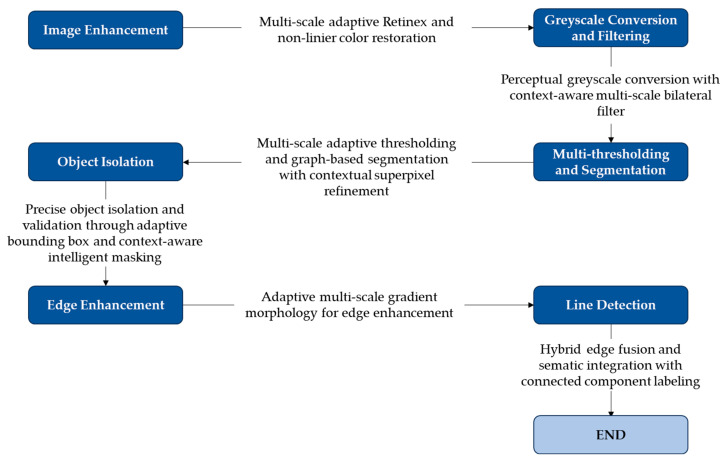
Precision ice detection on power transmission lines.

The main objective of ice detection on power transmission lines is to identify power transmission line icing (PTLI) images and isolate them from the background. After the ice-covered transmission lines are detected, the next step is determining the top and bottom boundary lines on the PTLI. This ice information is very important to achieve high accuracy in ice thickness measurement on transmission lines. If there is an error in determining the line on the formation, then the ice thickness measurement will also be inaccurate. This section provides the detailed method used to identify ice formations on power transmission lines utilizing image processing and machine vision technologies. The framework is divided into six stages. Each stage contains certain procedures and methods to improve image quality, improve and optimize the ice formation detection and segmentation process. The block diagram of enhanced image segmentation and edge detection using improved multi-scale Retinex and advanced morphological operations is shown in Figure 3.

In stage 1, image enhancement using multi-scale adaptive Retinex and non-linear color restoration techniques are applied. Input image preparation, initialization, multi-scale adaptive Retinex, non-linear color restoration with perceptual color enhancement and normalization, and advanced contrast enhancement with local adaptive techniques are composed in stage 1. In this stage, high-quality images of the power transmission line are captured and preprocessed to remove noise, correct distortions, and adjust the resolution. Multi-scale adaptive Retinex techniques are used to enhance lighting variations, followed by non-linear color restoration to improve contrast and visibility of ice formations on the transmission line. In stage 2, perceptual grayscale conversion with context-aware multi-scale bilateral filtering is applied. This stage consists of converting to grayscale with perceptual preserving conversion, dynamic parameter adjustment with context-aware analysis, multi-scale bilateral filtering with adaptive guided filtering and edge-aware weighting, and multi-resolution analysis with structural similarity. In this stage, grayscale conversion with luminance preservation and multi-scale bilateral filtering are applied to enhance edge details while reducing noise. The edge preservation technique ensures the clarity of ice and transmission lines. In stage 3, multi-scale adaptive thresholding and graph-based segmentation with contextual superpixel refinement are applied. Multi-Otsu thresholding with spatially adaptive methods, graph-based segmentation with geometric constraints, superpixel generation with contextual integration, and enhanced connectivity operations (dilation, closing) with topological analysis are composed in stage 3. In this stage, the system applies multi-Otsu thresholding and connected graph analysis to separate ice from the background, where superpixels and morphological changes help maintain connectivity.

In stage 4, context-adaptive intelligent masking and adaptive bounding box cropping for proper object isolation and validation are performed. This stage consists of adaptive cropping to bounding boxes with shape analysis, selective object removal with contextual awareness, and adaptive mask application with intelligent masking techniques. This stage of adaptive cropping and selective object removal isolates ice formations on the transmission line. In stage 5, adaptive multi-scale gradient morphology for edge enhancement with Canny–Laplacian fusion and sharpening are applied. Gradient morphology with enhanced feature extraction, chosen edge detection method (Canny combined with Laplacian) with adaptive thresholding, edge fusion, morphological sharpening with structural preservation, and pyramid-based multi-scale enhancement with adaptive blending are composed in this stage. This stage focuses on edge enhancement using gradient morphology and a combination of Canny and Laplacian edge detection, with edge fusion and multi-scale processing to improve clarity. In stage 6, hybrid edge fusion and semantic integration with connected component labeling are applied. This stage consists of hybrid edge fusion with semantic edge integration, advanced connected component labeling with region-based analysis, and contour drawing with contextual information and interactive visualization. This stage is used for line detection and determination of the upper and lower lines of the ice layer on the transmission line.

#### 2.2.1. Image Enhancement

This section details the image quality enhancement process for accurate PTLI detection, including the specific equations used in the process. The process begins with the preparation of the input image, which is captured using a binocular camera. Preprocessing stages consisting of noise removal and distortion correction are applied to improve image clarity, followed by resolution adjustment for uniformity in subsequent analysis. The image is resized to a uniform resolution using the following equations:

(1) $$I_{resize}\left(x^{\prime },y^{\prime }\right)=I_{corrected}\left(\frac{x\cdot W^{\prime }}{W},\frac{y\cdot H^{\prime }}{H}\right)$$

This equation adjusts an image’s resolution by mapping pixel coordinates from the original resolution $\left(W,H\right)$ to a new resolution $\left(W^{\prime },H^{\prime }\right)$. To obtain the pixel value at $\left(x^{\prime },y^{\prime }\right)$ in the resized image, the equation maps the coordinates $\left(x,y\right)$ from the original image to their new positions $\left(\frac{x\cdot W^{\prime }}{W},\frac{y\cdot H^{\prime }}{H}\right)$ in the resized image. It also analyzes the environment where the image is taken, including aspects like lighting or other easily recognizable items. The multi-scale adaptive Retinex technique is subsequently utilized to process the image in multi-scale in the sense that visibility in a number of scales of the PTLI is enhanced due to better control of the illumination changes. At this stage, the image will be contrast-enhanced by the following mathematical equation:

(2) $$I_{enhanced}\left(x,y\right)=\sum_{l=1}^{L}w_{l}\left[\log\left(I_{l}\left(x,y\right)\right)-\log\left(G_{\sigma l}⊗I_{l}\left(x,y\right)\right)\right]$$

This equation is part of the Retinex enhancement process, which improves image contrast by adjusting different scales. It produces the final enhanced image $I_{enhanced}\left(x,y\right)$ by combining images at various scales $I_{l}\left(x,y\right)$ with weighted Gaussian blurs $G_{\sigma l}$. The logarithmic difference, $\log\left(I_{l}\left(x,y\right)\right)-\log\left(G_{\sigma l}⊗I_{l}\left(x,y\right)\right)$, highlights contrast and details by emphasizing features in the image. The logarithmic difference between the original and blurred images enhances contrast and details.

Non-linear color restoration follows, adjusting colors to enhance visibility, as described by the equation:

(3) $$I_{perceptual}\left(x,y,c\right)=HVM\left(I_{chromatic}\left(x,y,c\right)\right)$$

The equation adjusts image colors for better balance and perceptual accuracy. $I_{chromatic}\left(x,y,c\right)$ is the image with corrected color intensity and gamma. HVM simulates human color perception, producing $I_{perceptual}\left(x,y,c\right)$, where colors are adjusted to match human visual sensitivity. Each pixel at coordinates $\left(x,y\right)$ and color channel c now reflects colors adjusted for better perceptual accuracy. The final steps in this stage include normalization to balance intensity levels and local contrast enhancement to further improve the visibility of PTLI details, ensuring optimal image preparation for grayscale conversion and bilateral filtering in the next stage. This stage uses contrast-limited adaptive histogram equalization (CLAHE, represented by the equation as follows:

(4) $$I_{final}\left(x,y\right)=CLAHE\left(I_{normalized}\left(x,y\right),clipLimit,tileGridSize\right)$$

**Figure 4:**
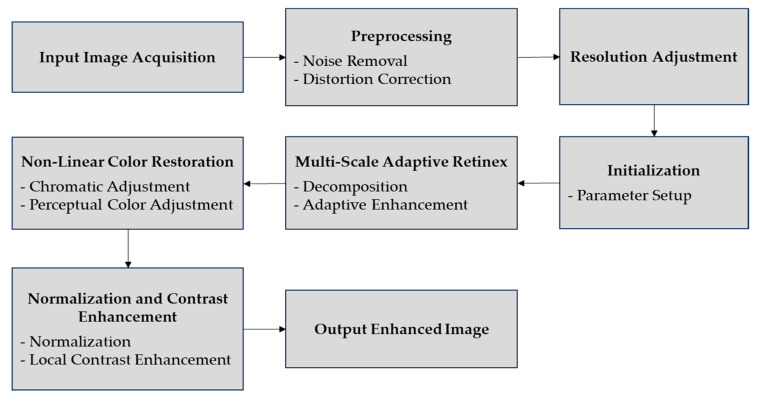
The block diagram of image enhancement.

The equation outlines the final enhancement using contrast-limited adaptive histogram equalization (CLAHE). $I_{normalized}\left(x,y\right)$ is the normalized image, with pixel intensities scaled for uniform brightness. clipLimit prevents noise by clipping the histogram, and tileGridSize divides the image into tiles for localized contrast adjustment. $I_{final}\left(x,y\right)$, shows improved local contrast and clarity, aiding in accurate ice detection on power transmission lines. Figure 4 shows the block diagram of image enhancement.

#### 2.2.2. Grayscale Conversion and Bilateral Filtering

The second stage is the perceptual grayscale conversion with context-aware multi-scale bilateral filtering. This stage starts with a grayscale conversion to make sure the image’s luminance remains high enough for the PTLI and background to retain high contrast. The following is the equation used in converting the image to grayscale:

(5) Y=0.2126⋅R+0.7152⋅G+0.0722⋅B

where R, G, and B represent the red, green, and blue color components of the image, respectively. This formula computes the grayscale value Y as a weighted sum of the RGB components, with the weights (0.2126, 0.7152, and 0.0722) reflecting the human eye’s varying sensitivity to different colors. This stage is followed by dynamic parameter adjustment, where the bilateral filter parameters are adjusted according to the specific characteristics of the image, emphasizing edge enhancement and noise reduction. The filter smooths while preserving edges using spatial and range Gaussian functions. Here, $I\left(x\right)$ is the filtered intensity, S is the spatial neighborhood, then $\left\|x-x_{i}\right\|$ and $\left\|I\left(x\right)-I\left(x_{i}\right)\right\|$ represent distances and intensity differences, respectively. Parameters $\sigma_{s}$ and $\sigma_{r}$ control spatial and range smoothing, with $W_{p}$ as the normalization factor.

(6) $$I\left(x\right)=\frac{1}{W_{p}}\sum_{x_{i}\in S}I\left(x_{i}\right)\cdot e^{-\frac{\|x-x_{i}{\|}^{2}}{2\sigma_{s}^{2}}}\cdot e^{-\frac{\|I\left(x\right)-I\left(x_{i}\right){\|}^{2}}{2\sigma_{r}^{2}}}$$

This method uses multi-scale bilateral filtering, which allows for preserving details in both fine and coarse PTLI structures. The multi-scale decomposition is performed using the following:

(7) $$I_{L}\left(x\right)=G_{\sigma }*I\left(x\right)$$

In this context, $I_{L}\left(x\right)$ is the low-pass filtered image at scale σ, obtained by convolving the image with a Gaussian kernel $G_{\sigma }$. The convolution operation (*) smooths the image, while guided filtering refines it while preserving edges.

(8) $$I_{q}\left(x\right)=a\left(x\right)\cdot I\left(x\right)+b\left(x\right)$$

In this process, $I_{q}\left(x\right)$ is the refined image from the guided filter, which preserves edges using the guidance image $I\left(x\right)$. Coefficients $a\left(x\right)$ and $b\left(x\right)$ are computed to minimize local differences between the input and output images. Multi-scale decomposition with Gaussian filtering is used to process images at different resolutions, and guided filtering further refines them based on the guide image. Adaptive guided filtering further improves this detail preservation by modifying the filtering process based on local image characteristics. Finally, edge-aware weighting and multi-resolution analysis use the Structural Similarity Index (SSIM) to ensure comprehensive enhancement:

(9) $$SSIM\left(x,y\right)=\frac{\left(2\mu_{x}\mu_{y}+C_{1}\right)\left(2\sigma_{xy}+C_{2}\right)}{\left(\mu_{x}^{2}+\mu_{y}^{2}+C_{1}\right)\left(\sigma_{x}^{2}+\sigma_{y}^{2}+C_{2}\right)}$$

**Figure 5:**
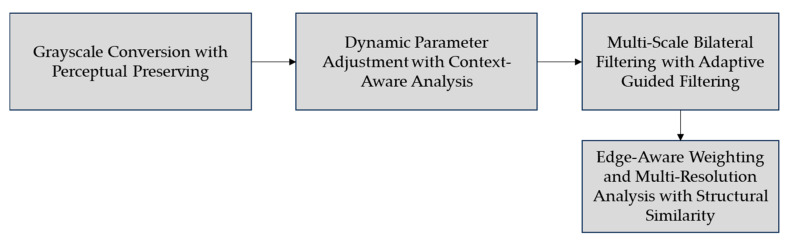
The block diagram of grayscale conversion and bilateral filtering.

In this context, $\mu_{x}$ and $\mu_{y}$ are the mean intensities of images x and y, $\sigma_{x}^{2}$ and $\sigma_{y}^{2}$ are their variances, and $\sigma_{xy}$ is their covariance. Constants $C_{1}$ and $C_{2}$ stabilize the division. The Structural Similarity Index (SSIM) uses these parameters to measure image similarity based on luminance, contrast, and structure. Figure 5 shows the block diagram of grayscale conversion and bilateral filtering.

#### 2.2.3. Thresholding and Segmentation

In stage 3, multi-Otsu thresholding with the spatial adaptive technique is applied to obtain more than one threshold. The object can be easily separated from transmission lines and other background features. This stage helps with the segmentation process because it identifies regions that might develop ice accumulation. The segmentation is further refined through a graph-based approach, where a graph is constructed with nodes representing pixels or superpixels and edges describing the relationships between them. The method calculates multiple thresholds ($T_{1},\;T_{2},\;\ldots \;,\;T_{n}$) and evaluates within-class variance to refine the segmentation.

(10) $$\sigma_{w}^{2}=\sum_{i=1}^{n}\omega_{i}\sigma_{i}^{2}$$

**Figure 6:**
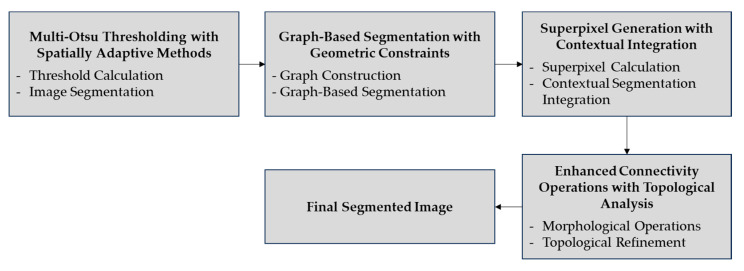
The block diagram of thresholding and segmentation.

Here, $\omega_{i}$ is the class probability, and $\sigma_{i}^{2}$ is the class variance. The goal is to minimize $\sigma_{w}^{2}$ to find optimal thresholds. Figure 6 shows the block diagram of thresholding and segmentation.

Once thresholds are set, segmentation isolates potential ice regions.

(11) $$R_{i}=\left\{\left(x,y\right)|T_{i-1}<I\left(x,y\right)\leq T_{i}\right\}$$

where $T_{0}=0$ and $T_{n}=255$. $I\left(x,y\right)$ is the pixel intensity at $\left(x,y\right)$. This method segments the image into regions based on these thresholds. Graph-based segmentation with geometric constraints involves constructing a graph representing the image and performing segmentation with geometric constraints for accurate delineation. Geometric constraints are then applied to accurately delineate PTLI. Superpixels are created to simplify this process, and contextual integration improves segmentation efficiency by focusing on areas with potential ice. Minimize the normalized cut as follows:

(12) $$minimize\frac{cut\left(S_{1},S_{2},\;\ldots ,\;S_{k}\right)\;}{assoc\;\left(S_{1},S_{2},\;\ldots ,\;S_{k}\right)}$$

where the cut is defined as follows:

(13) $$cut\left(S_{1},S_{2},\;\ldots ,\;S_{k}\right)=\sum_{i=1}^{k}\sum_{j\in S,l\notin S_{i}}w\left(e_{jl}\right)$$

The cut measures the total weight of edges separating segments $S_{1},S_{2},\;\ldots ,\;S_{k}$. For each segment $S_{i}$, it sums the weights $w\left(e_{jl}\right)$ of edges connecting nodes within $S_{i}$ to nodes outside $S_{i}$, defined as follows:

(14) $$assoc\;\left(S_{1},S_{2},\;\ldots ,\;S_{k}\right)=\sum_{i=1}^{k}\sum_{j\in S}\sum_{l\in V}w\left(e_{jl}\right)$$

The association measures the total edge weight within each segment $S_{i}$ relative to the entire graph V, summing $w\left(e_{jl}\right)$ for edges connecting nodes in $S_{i}$ to all other nodes in V.

(15) $$w\left(e_{ij}\right)=\exp\left(-\frac{\|I_{i}-I_{j}{\|}^{2}}{2\sigma^{2}}\right)$$

where $I_{i}$ and $I_{j}$ are the intensities of nodes i and j, and σ is a scaling parameter. This method builds a graph using pixel intensities and edge weights to reflect differences, then applies normalized cuts for segmentation while considering geometric constraints. Superpixel generation with contextual integration simplifies the segmentation process by generating superpixels that focus on areas with potential ice and integrate contextual information for efficient segmentation. The superpixel calculation is as follows:

(16) $$d=\sqrt{{\left(\frac{\Delta L}{N}\right)}^{2}+{\left(\frac{\Delta a}{N}\right)}^{2}+{\left(\frac{\Delta b}{N}\right)}^{2}+{\left(\frac{\Delta x}{S}\right)}^{2}+{\left(\frac{\Delta y}{S}\right)}^{2}}$$

where ΔL, Δa, and Δb are color space differences, Δx and Δy are spatial differences, and N and N are normalization factors. Contextual similarity is as follows:

(17) $$R_{i}=\left\{S_{j}contextual\_similarity\left(S_{j},C\right)>\theta \right\}$$

where $S_{j}$ are superpixels, C is the contextual information, and θ is a threshold for similarity. Enhanced connectivity operations (dilation, closing) with topological analysis apply morphological operations to enhance connectivity in segmented regions, refining segmentation quality through topological analysis. Morphological operations are as follows:

Dilation:

(18) $$A⊕B=\left\{z|{\left(B\right)}_{z}\cap A\neq \emptyset \right\}$$

Closing:

(19) $$A\cdot B=\left(A⊕B\right)⊖B$$

where A is the image, B is the structuring element, ⊕ denotes dilation, and ⊖ denotes erosion. The refined segment is as follows:

(20) $$refinedSegment=\left\{R_{i}\left|size\;R_{i}\right.>\epsilon \right\}$$

where *ϵ* is a size threshold to remove small segments.

#### 2.2.4. Object Isolation and Validation

**Figure 7:**
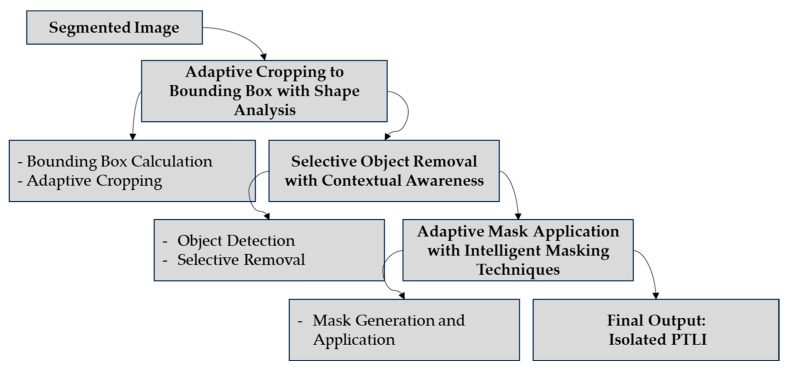
The block diagram of object isolation and validation.

In stage 4, the method is likely to target object isolation and its validation through adaptive cropping and shape analysis. Figure 7 shows the block diagram of object isolation and validation.

First, the size of the ice formations is determined by approximating bounding boxes of the detected regions depending on their shape so that the location can be determined correctly. This stage is succeeded by adaptive cropping, in which the image is cropped where necessary to increase the amount of identified ice and reject irrelevant areas. With the bounding box calculation using the shape analysis equation, let $R_{i}$ be the region i in the segmented image.

(21) $$BB_{i}=\left(\min\left(x_{i,j}\right),\;\min\left(y_{i,j}\right),\max\left(x_{i,j}\right),\max\left(y_{i,j}\right)\right)$$

Here, $\left(x_{i,j},y_{i,j}\right)$ are the boundary pixel coordinates of region $R_{i}$. The bounding box $BB_{i}$ is a rectangle enclosing $R_{i}$, defined by the min and max coordinates of these boundary pixels. Adaptive cropping is applied to focus on areas containing identified ice formations, effectively removing unnecessary background elements. Adaptive cropping equation,

(22) $$I_{c}=I\left(BB_{i}\right)$$

Here, I is the original image, $BB_{i}$ is the bounding box for the region of interest, and $I_{c}$ is the cropped image based on $BB_{i}$. The process isolates the relevant region from the original image by removing background elements. To further refine the analysis, non-ice objects are detected using contextual awareness to understand their spatial relationships and significance, followed by their selective removal to isolate only the relevant ice regions. Object detection with contextual awareness equation

(23) $$O_{i}=\left\{R_{i}|f(R_{i})=1\right\}$$

Here, $O_{i}$ is the set of objects for removal, and f determines if a region $R_{i}$ should be removed based on context. f returns 1 to remove and 0 to keep each region.

(24) Ir=IcOi

In this process, $I_{r}$ is the image after removing unwanted objects $O_{i}$ from the cropped image $I_{c}$, resulting in a clean image for further analysis. Next, intelligent masking techniques are employed, where masks are generated adaptively to isolate ice formations accurately, considering their contours and characteristics:

(25) $$M_{i}=g\left(I_{r},p\right)$$

In this context, $M_{i}$ is a binary mask for object i, generated by the function g using parameters p. These masks are then applied to the images to enhance the visibility of ice formations, ensuring clear and distinct isolation from the background. The mask application equation is as follows:

(26) $$I_{o}=I_{r}⊙M_{i}$$

In this context, $I_{o}$ is the image with isolated objects, where ⊙ denotes the application of the mask $M_{i}$ to the image $I_{r}$. Before masking, $I_{r}$ is the image with unwanted objects removed. The mask $M_{i}$ isolates desired objects, producing $I_{o}$ with clearly highlighted areas.

#### 2.2.5. Edge Enhancement

**Figure 8:**
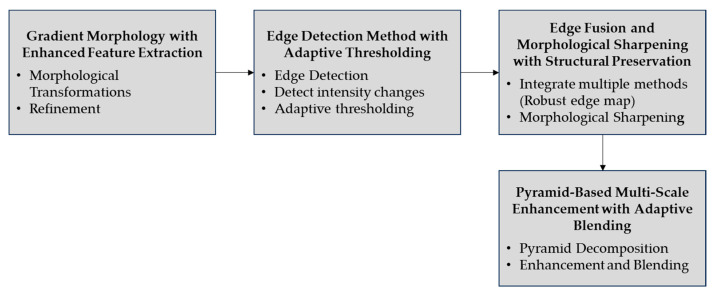
The block diagram of edge enhancement.

In stage 5, edge enhancement is performed through a sequence of advanced techniques aimed at refining the boundaries of the ice formation. Gradient morphology with enhanced feature extraction leverages mathematical morphology to process images based on shapes. Figure 8 shows the block diagram of edge enhancement.

This stage involves the refinement of detected edges by further sharpening and clarifying them, which includes smoothing and removing extraneous details to ensure well-defined edges and reduced noise. The equation for gradient morphology is as follows:

(27) $$G\left(x,y\right)=Dilatation\left(I\left(x,y\right)\right)-Erosion\left(I\left(x,y\right)\right)\;$$

where $G\left(x,y\right)$ is the gradient image derived from $I\left(x,y\right)$. Morphological operations, dilation, and erosion adjust image structure: Dilatation $I\left(x,y\right)$ expands the boundaries of regions of interest within the image, while Erosion $I\left(x,y\right)$ shrinks these regions. The edge detection method with adaptive thresholding employs gradient calculation, non-maximum suppression, and edge tracking by hysteresis to detect a wide range of edges with minimal noise. The equation for refinement is as follows:

(28) $$E_{r}=NMS\left(G\left(x,y\right)\right)\cdot Hysterisis\left(G\left(x,y\right)\right)$$

In image processing, $E_{r}$ is the refined edge map, obtained by applying non-maximum suppression (NMS) to $G\left(x,y\right)$ to thin edges, and edge tracking by hysteresis $Hysterisis\left(G\left(x,y\right)\right)$ to link and preserve significant edges.

Combining Canny and Laplacian methods ensures comprehensive edge detection and adaptive thresholding dynamically adjusts threshold values based on local image characteristics, maintaining accurate edge detection without losing essential details or introducing excessive noise. The equation for adaptive thresholding is as follows:

(29) $$E_{t}\left(x,y\right)=T_{adaptive}\left(E_{c}\left(x,y\right),\;E_{l}\left(x,y\right)\right)$$

$E_{t}\left(x,y\right)$ is the combined edge map formed by integrating $E_{c}\left(x,y\right)$ from the Canny method and $E_{l}\left(x,y\right)$ from the Laplacian method using adaptive thresholding $T_{adaptive}$. This method dynamically adjusts thresholds to capture significant edges while minimizing noise. Edge fusion and morphological sharpening with structural preservation integrate edges detected from multiple methods to create a robust edge map, compensating for each method’s weaknesses and applying dilation and erosion to sharpen edges while preserving structural integrity, thus enhancing clarity and definition without introducing artifacts. The equation for morphological sharpening is as follows:

(30) $$E_{s}\left(x,y\right)=Dilatation\left(E_{f}\left(x,y\right)\right)-Erosion\left(E_{f}\left(x,y\right)\right)$$

$E_{f}\left(x,y\right)$ is the fused edge map combining various detection methods. It is then refined to produce $E_{s}\left(x,y\right)$, the sharpened edge map. Pyramid-based multi-scale enhancement with adaptive blending involves decomposing the image into a pyramid of multiple scales, allowing for detailed analysis and enhancement at various resolutions. Scale-specific techniques such as contrast adjustment, sharpening, and noise reduction are applied at each pyramid level, and adaptive blending combines these enhancements smoothly, maximizing overall visual quality and detail. The equation for adaptive blending is as follows:

(31) $$I_{final}\left(x,y\right)=\sum_{i}w_{i}\cdot P_{i}^{\prime }\left(x,y\right)$$

In multi-scale image enhancement, $P_{i}^{\prime }\left(x,y\right)$ denotes the enhanced image at scale i after applying techniques like contrast adjustment and sharpening. These images are combined using blending weights $w_{i}$ to create $I_{final}\left(x,y\right)$, the final enhanced image, ensuring optimal visual quality.

#### 2.2.6. Line Detection

The final process of ice formation identification on transmission lines is line detection and visualization. A multi-scale merging of the edges is detected using an innovative edge fusion strategy to provide a complete edge map. There is a combined edge map here that is semantically enhanced through integration and used to strengthen the identification of the edges of the upper and lower ice structures. Advanced connected component labeling is applied to identify and label distinct ice regions in the edge map, followed by region-based analysis to refine these labeled components for greater precision. The contour detection method is then used to outline significant ice structures, and these contours are drawn with contextual information to improve interpretability. Finally, interactive visualization elements are introduced to allow dynamic exploration of contours and context, facilitating a more comprehensive understanding of the PTLI identification (top and bottom line). The equation for semantic enhancement is as follows:

(32) $$E_{s}\left(x,y\right)=E_{f}\left(x,y\right)\cdot S\left(x,y\right)$$

In ice detection, $E_{f}\left(x,y\right)$ is the fused edge map combining multiple scales and methods. This is enhanced to $E_{s}\left(x,y\right)$ using a semantic factor $S\left(x,y\right)$ to highlight and accurately represent ice-related edges. Advanced connected component labeling with region-based analysis labels connected components in the edge map, identifying distinct ice regions and refining them using region-based techniques. The equation for connected component labeling is as follows:

(33) $$L\left(x,y\right)=\sum_{j=1}^{M}C_{j}\left(x,y\right)$$

In connected component analysis, $L\left(x,y\right)$ labels each pixel for its connected component, $C_{j}\left(x,y\right)$ indicates the presence of the j−th component, and M represents the total number of detected components. Contour drawing detects and outlines significant ice structures with contextual details and interactive features. Final post-processing enhances image quality and smooths out artifacts. The equation for interactive visualization is as follows:

(34) $$V\left(x,y\right)=g\left(\partial R\left(x,y\right),\;C\left(x,y\right)\right)$$

**Figure 9:**
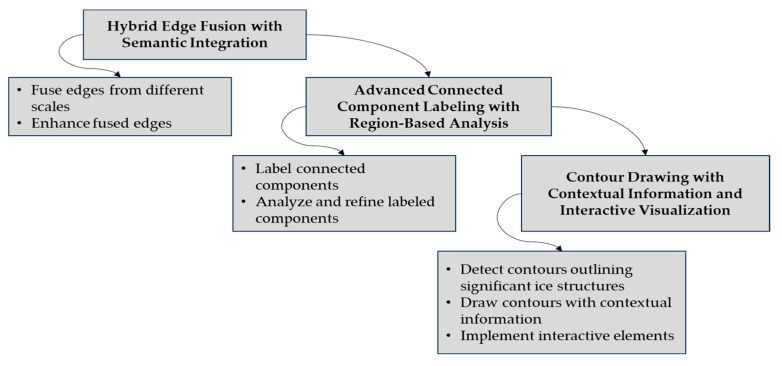
The block diagram of line detection.

In ice structure analysis, $\partial R\left(x,y\right)$ denotes contour points of significant formations. The interactive visualization $V\left(x,y\right)$, combines these contours with contextual data $C\left(x,y\right)$ using function $g\left(\cdot \right)$, enhancing interpretability and exploration of the ice structures. Figure 9 shows the block diagram of line detection.

## 3. Results and Discussion

The results of each stage in the proposed image processing workflow are presented and analyzed in this section. The proposed method consists of six sequential stages that progressively refine and segment power transmission line images to enable accurate identification of ice formations. Every stage integrates state-of-the-art procedures to solve several issues, including noise elimination problems, color processing, binarization issues as well as edge detection, which are crucial for precise ice monitoring at the transmission lines.

### 3.1. Individual Stage Performance in PTLI Identification

#### 3.1.1. Image Enhancement Performance

**Figure 10:**
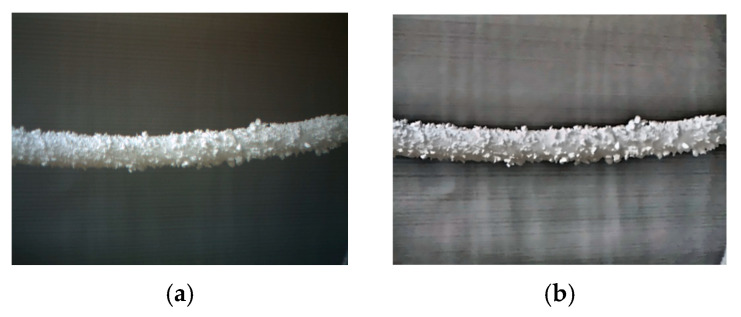
The enhanced image using multi-scale adaptive Retinex and non-linear color restoration method: (**a**) The original image; (**b**) The enhanced image.

The image enhancement process is very important for enhancing the quality of PTL images. Through a multistage, including input image preparation, parameter adjustment, and multi-scale adaptive techniques, this stage aims to optimize the contrast, clarity, and visual details of the images. Processes such as noise removal, distortion correction, and resolution adjustment aim to ensure that the resulting images meet the standards for further analysis. In addition, the use of multi-scale adaptive Retinex and non-linear colors are used to increase the contrast and improve color accuracy in order to unmask the ice and distinguish it from its background. Thus, this stage produces images that are not only high quality but also ready for further processing to detect and map ice on transmission lines with higher accuracy. Figure 10 shows the results of the proposed method for image enhancement.

In the image enhancement stage of the process, the results show a good improvement in image quality, which is very important for detecting ice formation on power transmission lines. Input image preparation plays a key role in ensuring image clarity from the start, where noise removal and distortion correction are effective in preserving the surface details of the icy transmission line. This step reduces interference from weather conditions such as rain or snow, which can affect visual quality, such as lighting in the image. Next, Multi-Scale Adaptive Retinex provides improvements in lighting by decomposing the image into multiple scales to adaptively adjust for lighting variations. This method improves the contrast and visibility of PTLI, making the contours of the PTLI easier to recognize without losing important details transmission line.

Next, non-linear color restoration with perceptual color enhancement successfully improves color sharpness, which helps distinguish the ice-covered area from the transmission line itself. The chromatic adjustment process restores the original color, while the perceptual color adjustment provides improvements in brightness and color contrast, making the PTLI area more visible. Finally, normalization and local adaptive contrast enhancement enhance local details in relevant areas, balance the overall intensity, and provide clearer contrast between the ice-covered transmission channel and other objects. Based on these steps, the resulting image has better clarity, helping subsequent processing steps in more accurate ice identification and segmentation.

#### 3.1.2. Grayscale Conversion and Bilateral Filtering Performance

**Figure 11:**
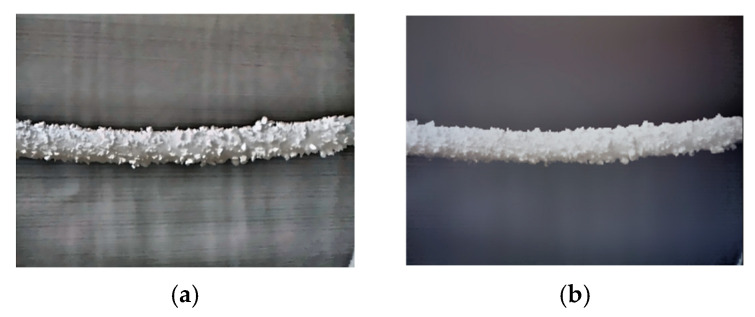
The perceptual grayscale conversion with context-aware multi-scale bilateral filtering: (**a**) The enhanced image; (**b**) The filtered image.

The grayscale conversion and bilateral filtering stage play an important role in providing the clarity and visual sharpness of the PTLI, which is crucial for further analysis. This stage preserves the original luminance so that the ice-covered transmission line remains visible and easily distinguishable by perceptual grayscale image conversion. This stage is completed by dynamic adjustment of the bilateral filter based on the image context. Moreover, the described multi-scale bilateral filtering and the adaptive guided filtering allow the adjustment at such scales, thus allowing the visibility of fine and coarse details. Edge-aware weighting and multi-resolution analysis-based methods provide an additional layer in preserving the integrity of the edges from the PTLI structure. Figure 11 shows the result of perceptual grayscale conversion with context-aware multi-scale bilateral filtering.

The enhanced image (Figure 11a) improves the visibility of transmission lines and ice formations under various lighting conditions, while grayscale conversion and bilateral filtering (Figure 11b) preserve important contrast, reduce noise, and maintain sharp edges. In this stage, the results obtained show the effectiveness of the process in maintaining important details in the image, especially in the ice structure on the transmission line. The conversion to grayscale with a perceptual preserving conversion process ensures that the conversion to grayscale maintains perceptual luminance so that the visual characteristics of the PTLI image are clearly maintained. This helps distinguish ice formations from the background without losing important information. Also, dynamic parameter adjustment with a context-aware analysis mechanism encompasses a bilateral filter for adjusting both the parameters of the filter dynamically in relation to the characteristics of the image. This is useful for stabilizing edges and minimizing noise that significantly clarifies the image when pointing to certain features in the PLTI.

In addition, the application of multi-scale bilateral filtering with adaptive guided filtering enhances the process with multi-scale, which maintains fine and coarse details in the ice structure, adaptively adjusting the filter based on local characteristics of the ice and transmission line to maintain image integrity. Edge-aware weighting and multi-resolution analysis techniques with structural similarity metrics also show good results in maintaining the clarity of ice and cable edges. With an edge-preserving approach, images retain important elements despite noise-reducing filtering. The use of multi-resolution analysis enhances the resulting image sufficiently and makes a strong platform for further analysis for the identification of ice and its segmentation.

#### 3.1.3. Thresholding and Segmentation Performance

The thresholding and segmentation stage focuses on separating the PTLI from the background and other components. This stage provides an accurate mapping between the ice-covered transmission line and the background by applying multi-Otsu thresholding and spatial adaptive methods. Graph-based segmentation methods with geometric constraints and superpixel integration further strengthen object separation through graphical representation and context-based segmentation. Finally, morphological operations such as dilation and closing with topological analysis help preserve the connectivity of the ice structure, resulting in a more defined segmentation ready for further analysis.

**Figure 12:**
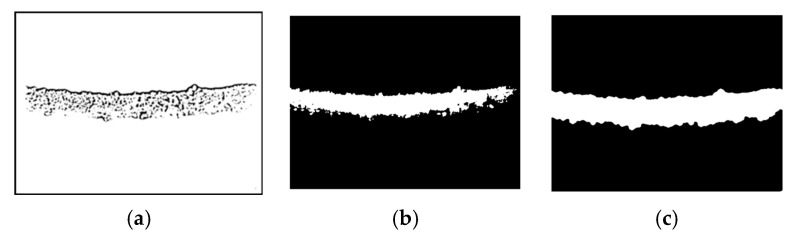
The multi-scale adaptive thresholding and graph-based segmentation with contextual superpixel refinement result: (**a**) The adaptive thresholding; (**b**) The multi-Otsu thresholding; (**c**) The proposed method.

Figure 12a,b shows the results of adaptive and multi-Otsu thresholding, while Figure 12c shows the proposed method. Figure 12 illustrates the comparative effectiveness of three segmentation methods for detecting ice on power transmission lines. In the thresholding and segmentation stage, the results obtained show a significant improvement in the separation of ice areas from the background and other components in the image. The use of multi-Otsu thresholding with the spatial adaptive method successfully calculates several thresholds to separate ice-covered transmission lines and other background elements more accurately. This segmentation results in a clearer mapping of the ice area, making it easier to identify ice in the next stage.

In addition, the integration of graph-based segmentation with geometric constraints builds a graphical model of the image where vertices require superpixels while edges refer to connections between vertices. This approach guarantees a clear distinction between the regions of ice and the background so that other significant objects in the image are differentiated well. Another role of superpixel generation with contextual integration in the segmentation system is that it effectively reduces the segmentation difficulty by generating superpixels that only target pixels as potential ice areas. The final operation is connectivity operations with topological analysis for illumination. The topological analysis performed at this stage allows for the smoothing of the outline and enforces the segmentation of the ice structure. Overall, this process enables better segmentation, which then helps when making and validating vectors in other subsequent steps.

#### 3.1.4. Object Isolation and Validation Performance

**Figure 13:**
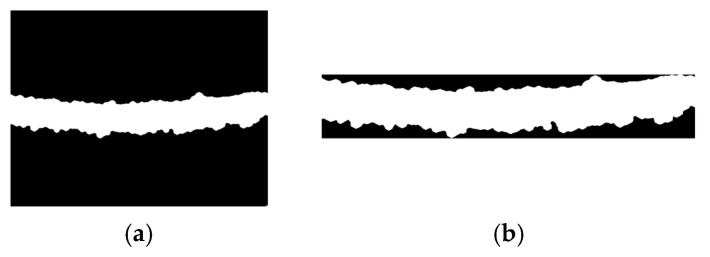
The precise object isolation and validation through adaptive bounding box cropping and context-aware intelligent masking: (**a**) The segmentation image; (**b**) The object isolation.

The object isolation and validation stage focuses on separating and highlighting the PTLI areas from other elements in the image for more effective analysis. Figure 13 shows this method’s effectiveness by cropping the image to the bounding box.

The results obtained in the object isolation and validation stage proved to be better in terms of separation of PTLI regions, where further analysis has been made much easier. The adaptive cropping to bounding box with shape analysis process allows the adaptive determination of the bounding box so that areas with ice formations are detected with precision and the image can be focused on relevant areas containing PTLI. This process reduces unnecessary regions of the image, thus increasing the efficiency of subsequent analysis. In addition, selective object removal with contextual awareness is able to identify all non-ice objects that might hinder the identification process, such as shadows or other irrelevant objects. This way, the ice areas are very well-separated, and no influence is exerted on them, which may lead to inaccurate identification of the ice areas. The last process, adaptive mask application with intelligent masking techniques, produces a mask that adaptively separates ice formations accurately from the background. All these stages successfully improve the focus on significant PTLI regions.

#### 3.1.5. Edge Enhancement Performance

**Figure 14:**
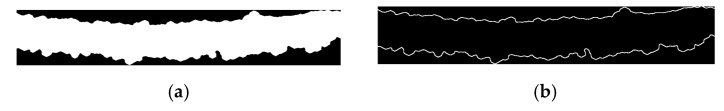
The adaptive multi-scale gradient morphology for edge enhancement with Canny–Laplacian fusion and sharpening: (**a**) The object isolation; (**b**) The edge detection.

The edge enhancement stage focuses on improving the sharpness and clarity of the ice formation edges on the transmission line. The process comprehensively enhances the ice edge details by applying gradient morphology and Canny–Laplacian combined edge detection methods enhanced by adaptive thresholding. The edge fusion and morphological sharpening processes ensure detailed edge detection results without compromising the ice structure. The pyramid-based multi-scale enhancement further improves edge detection at multiple scales to accommodate edge analysis at higher levels of precision. As illustrated in Figure 14 above, this process improves the ice formation edges’ accuracy for identification and analysis in power line maintenance purposes. Shown in Figure 14 is the use of the adaptive multi-scale gradient morphology integrated with Canny–Laplacian fusion and sharpening processes in boosting edge enhancement in the identification of ice on power transmission lines. The isolation of the objects is achieved, as illustrated in Figure 14a, thus providing a good ground for subsequent edge enhancement. Figure 14b highlights how the proposed method produces a sharp and detailed edge map, with gradient morphology refining significant transitions and minimizing minor artifacts.

#### 3.1.6. Line Detection Performance

**Figure 15:**
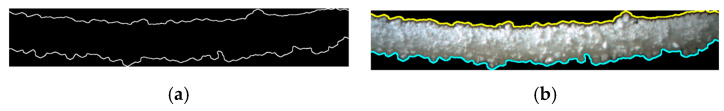
The results of line detection achieved through comprehensive multi-scale edge detection and visualization with a semantic enhancement: (**a**) The edge detection; (**b**) The line detection.

The last stage involves precisely defining the top and bottom boundaries of icing at power transmission lines, which is important for 3D icing thickness measurement. Fine and coarse edges that determine the object shape are visualized by employing multi-scale edge detection and visualization. Hybrid edge fusion refines the results further to differentiate between different regions of ice with semantic edge detection and connected component labeling. Contour detection and interactive visualization assist in advancing the understanding of the results and navigating them. This way of dealing with the problem guarantees the identification of the main areas and types of ice concentration, the thickness of which will need to be removed, thus reflecting the real situation as closely as possible. Figure 15 presents these line detection results. Line detection was performed based on the edges detected in the second phase, as described in Section 3.

The findings of this research show that using multi-scale edge detection and visualization incorporated with semantic enhancement is important for PTLI. This approach greatly improves the identification of the upper and lower edges of the PTLI and is critical for accurate depth measurements in 3D imaging. Figure 15b represents the final edge-detected image where connected component labeling segregates these areas to a greater extent. The application of yellow color for the top line and cyan for the bottom line makes it easier to distinguish and estimate the ice concentration on the map based on its color gradient pattern. This is practiced using multi-scale edge detection that detects minor features and hybrid edge fusion that captures major features, thereby detecting boundaries clearly for evaluation.

### 3.2. Quantitative Evaluation

Overall, the results of PTLI identification are very dependent on its segmentation process, which incorporates image enhancement, filtering and thresholding. Segmentation errors directly affect the shape and the edges of the ice formations, which must be detected in order to acquire accurate thickness measurements. The method’s performance is rigorously evaluated using metrics like accuracy, precision, sensitivity/recall, and specificity. Two evaluation methods are employed: one assesses each stage (image enhancement, filtering, and segmentation) individually, while the other evaluates the overall PTLI identification scheme using comprehensive metrics. This dual approach ensures a thorough assessment of both individual components and the integrated system’s effectiveness and reliability. Table 1 summarizes the evaluation metrics used.

Table 1 is a confusion matrix used to evaluate the performance of the power transmission line icing (PTLI) identification method. This matrix compares the model’s predictions with the actual, real-world conditions, known as ground truth, to assess its accuracy. It categorizes predictions as either PTLI (indicating the presence of icing) or Not PTLI (indicating the absence of icing). The effectiveness of the proposed method in accurately identifying PTLI or non-PTLI can be determined by analyzing these metrics, and this provides insight into its overall reliability and precision.

The table categorizes the outcomes of the method’s predictions compared to the ground truth into four types:

- True Positive (TP): the method correctly identifies the presence of icing (PTLI).
- True Negative (TN): the method correctly identifies the absence of icing (Not PTLI).
- False Positive (FP): the method incorrectly predicts icing when there is none.
- False Negative (FN): the method fails to detect icing when it is actually present.

The counts of True Positives (TPs), True Negatives (TNs), False Positives (FPs), and False Negatives (FNs) are used to calculate key performance metrics like accuracy, precision, recall, and specificity. These metrics quantify the effectiveness of the PTLI identification method: accuracy measures overall correctness, sensitivity evaluates the method’s ability to identify actual ice formations, precision assesses the accuracy of positive predictions, and specificity indicates how well the method distinguishes between ice and non-ice regions. Together, these metrics provide a comprehensive evaluation of the method’s performance.

- Accuracy is computed as the ratio of correctly identified line icing on power transmission (both true positives and true negatives) to the total number of instances, reflecting the overall correctness of the identification process:

(35) $$Accuracy=\frac{TP+TN}{TP+TN+FP+FN}$$

- Sensitivity/recall measures the proportion of actual ice formations correctly identified by the method, highlighting its effectiveness in detecting true positives:

(36) $$Sensitivity=\frac{TP}{TP+FN}$$

- Precision indicates the proportion of true positive detections among all positive identifications made by the method, assessing the accuracy of positive predictions:

(37) $$Precision=\frac{TP}{TP+FP}$$

- Specificity evaluates the method’s ability to correctly identify non-ice regions, indicating its proficiency in distinguishing between ice and other elements:

(38) $$Specificity=\frac{TN}{TN+FN}$$

These evaluation metrics collectively provide a comprehensive assessment of the PTLI identification scheme’s performance, ensuring that the detection and isolation of ice formations are accurately measured.

#### 3.2.1. Performance Evaluation Through Quantitative Metrics for Each Stage

The performance of each stage in the proposed PTLI identification scheme is rigorously evaluated using sensitivity validation metrics, which measure the method’s ability to correctly identify actual ice formations (True Positives). Seven distinct methods were tested to assess the robustness of the integration between image processing stages, including image enhancement (E), filtering, thresholding (F), and multi-threshold segmentation (M-S). These methods include:

- PTLI identification using image enhancement, filtering, and multi-threshold segmentation;
- PTLI identification using image enhancement and multi-threshold segmentation;
- PTLI identification using multi-threshold segmentation alone;
- PTLI identification using filtering and multi-threshold segmentation;
- PTLI identification using image enhancement and filtering;
- PTLI identification using filtering alone;
- PTLI identification using image enhancement alone.

The method’s effectiveness was tested using the sensitivity equation on 30 randomly selected PTLI images to analyze each stage of the identification process. Table 2 shows the sensitivity results for image enhancement, filtering, and multi-threshold segmentation, providing values and percentages that demonstrate the effectiveness of these PTLI identification processes. The (∨) sign indicates that the method is used, and the (-) sign indicates that the method is not used. Table 2 shows the sensitivity validation results for different image processing methods in PTLI identification. The method involving image enhancement, filtering, and multi-threshold segmentation had a relatively high sensitivity of 90.18%, a highly accurate system for detecting ice formations. The methods that did not use filtering or image enhancement had trends toward lower sensitivity of 77.98% and 73.69%, which suggests their necessity. The absence of multi-threshold segmentation led to even lower sensitivities, underscoring the crucial role of segmentation in accurate ice detection.

**Figure 16:**
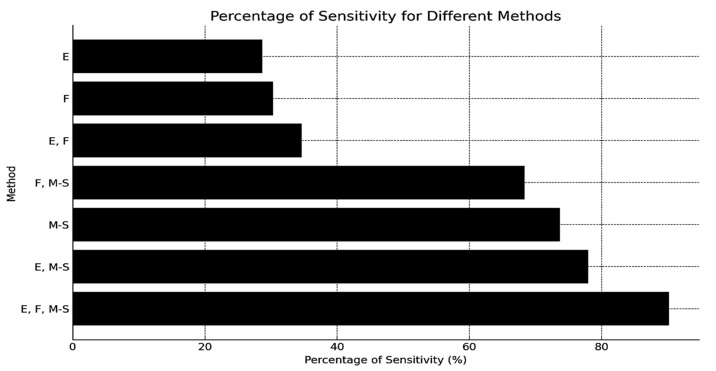
The percentage of sensitivity for different methods.

Figure 16 shows the sensitivity percentages for different methods, highlighting that the highest sensitivity (90.18%) is achieved when combining image enhancement (E), filtering (F), and multi-threshold segmentation (M-S). Individual methods like E and F have lower sensitivity levels. Figure 16 provides an example of segmentation results from a system that does not integrate all three methods, illustrating the reduced effectiveness without their combination.

**Figure 17:**
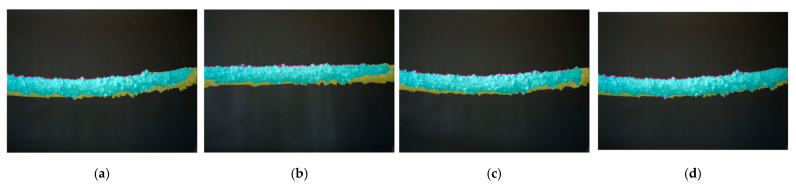
The example of segmentation results of a system that does not integrate the three methods: (**a**) Image enhancement and multi-thresholding; (**b**) Multi-thresholding; (**c**) Filter and multi-thresholding; (**d**) Image enhancement, filter and multi-thresholding.

Figure 17 illustrates the segmentation results of a system that lacks the integration of image enhancement, filtering, and multi-threshold segmentation. The cyan area shows true positives, where icing is correctly detected, while magenta indicates false positives, and yellow represents false negatives. The black area denotes true negatives. This analysis reveals the system’s limitations in accurately distinguishing between icing and non-icing areas without the full integration of all three methods. It underscores the importance of a multistage (enhancement, filter, and multi-thresholding) approach for maximizing sensitivity and accuracy in PTLI identification, highlighting that omitting any stage reduces overall effectiveness.

#### 3.2.2. Quantitative Evaluation of PTLI Identification Scheme Performance

**Figure 18:**
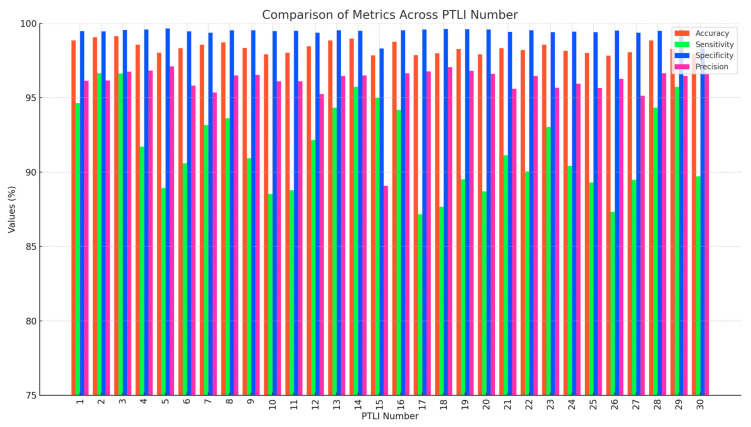
Comparison of accuracy, sensitivity, specificity, and precision (in %) across different PTLI numbers.

In this research, the proposed power transmission line icing (PTLI) identification scheme is quantitatively assessed. This is assessed by comparing the values of accuracy, precision or the measurement of accuracy, sensitivity or recall, which refers to the rate at which it correctly identifies the ice formations, and specificity, which measures the proportion of negatives that are not classified as positives. The advantages and challenges revealed in this paper enlighten the factors that can affect the method positively or negatively, with reference to the segmentation process. As depicted in Figure 18, different curves are plotted to represent the performance of different examples of PTLI models where different colors are used for each model. The results discussed in this evaluation approve the scheme for realistic applications.

Figure 18 provides a comparative analysis of four key performance metrics, including accuracy, sensitivity, specificity, and precision across different PTLI instances. The proposed PTLI identification method shows high accuracy (from 97.8% to 99.1%) and specificity (often exceeding 99.5%), indicating its reliability in correctly identifying ice formations and distinguishing them from non-ice regions. The sensitivity decreases between 87.1% and 96.6%, which means that under certain circumstances, some real ice formations remain undetected. The sensitivity remains close to ideal (from 89.0% to 97.1%), although a decrease in the noise level may occur. In general, the considered method is quite efficient and accurate, but further improvements may be needed to increase the sensitivity if it is to be applied in a wider range of conditions. Thus, the experience allows us to highlight the strengths and problems of the method that require improvement and also to confirm its readiness for practical applications.

**Figure 19:**
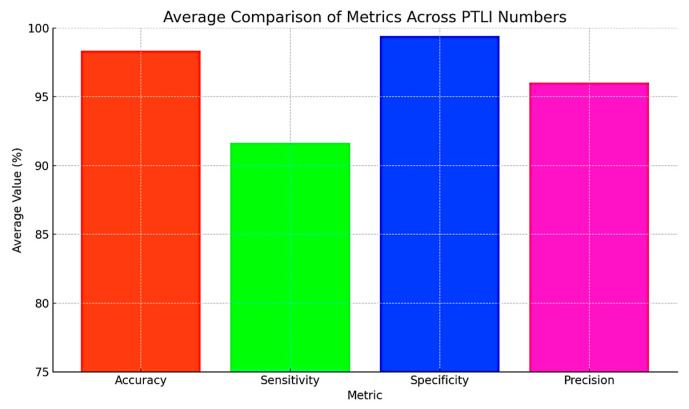
The bar chart represents the average values of accuracy, sensitivity, specificity, and precision.

In Figure 19, a bar chart is used to compare the average values of accuracy, sensitivity, specificity, and precision in percentage format with data labels. These measurements clearly show that the PTLI identification scheme is quite efficient and reliable as its average accuracy is 98.35% and specificity is 99.42%, with an estimated average precision of 96.03%. Because the values extracted from the Figure 19 support the proposed method, they also clearly qualify the method in the correct identification of ice formations on transmission lines and the elimination of false alarms. However, with a slightly lower average sensitivity of 91.63%, the algorithm can be improved to detect all types of real ice formations under difficult conditions. In conclusion, it can be stated that the high accuracy, specificity, and precision confirm the applicability of the proposed method in practice and indicate opportunities for improvement.

### 3.3. Real-Time Processing Capabilities

This section presents the results of processing time per frame for each stage of the method (image enhancement, grayscale and filtering, segmentation, object isolation, edge detection, and line detection). To evaluate the real-time processing capability of the proposed method, this study conducted a performance test on a machine equipped with an Intel Core i5 processor and 32 GB RAM. Figure 10 shows the processing time in different stages and each frame.

**Figure 20:**
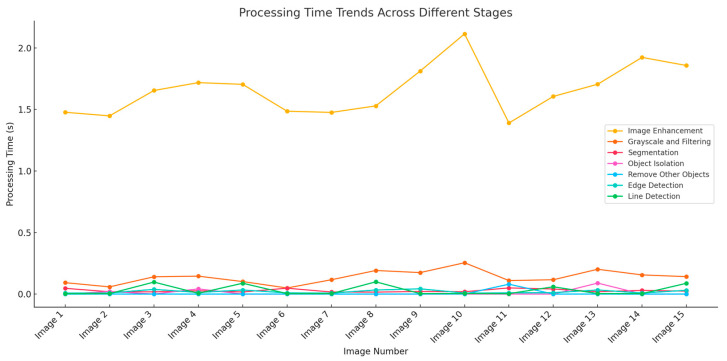
The processing time in different stages and each frame.

Based on Figure 20, image enhancement requires the longest processing time and faster processing than image enhancement in grayscale and filtering. Segmentation, object isolation, removal of other objects, edge detection, and line detection need a faster processing time than image enhancement and the filtering stage. It can be seen that their positions are below. The average processing time shows that image enhancement is by far the most time-consuming stage, with an average of 1.6599 s, which contributes the most to the total processing time of 1.8937 s. Grayscale and filtering are the next largest contributors, with an average of 0.1371 s, but are still relatively faster compared with image enhancement. Stages such as segmentation, object isolation, and edge detection have lower average times (ranging from 0.0059 to 0.0319 s), indicating that these stages have minimal impact on the total time. The average processing time per frame, including image enhancement, grayscale and filtering, segmentation, object isolation, edge detection, and line detection, is about 1.89 s. This processing time indicates that the proposed method is feasible for the application of real-time ice monitoring on transmission lines.

Regarding scalability, the proposed method can be adapted to monitor multiple transmission lines using a distributed processing framework. For large-scale networks, parallel processing across multiple devices or cloud-based processing can handle large amounts of image data. The application of the proposed method to UAVs offers an adaptable and portable system for monitoring transmission line icing. Through binocular cameras fitted in the UAVs, transmission line icing images can be captured in different surroundings effectively; thus, real-time ice detection is possible in worse climate conditions. The following research will focus on further improvements and actualization of optimizations on lightweight UAV platforms for broader applications. This method will increase UAV monitoring effectiveness in analyzing the transmission line status, which in turn will help to increase the safety of the process.

### 3.4. Application of PTLI Identification in Diverse Scenarios

**Figure 21:**
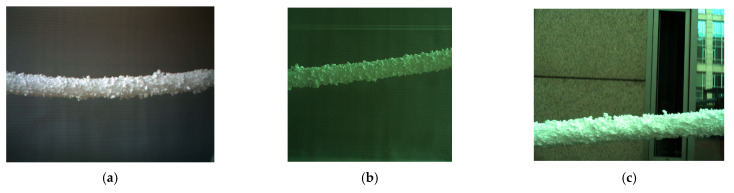
The tested images under diverse conditions: (**a**) PTLI 1; (**b**) PTLI 2; (**c**) PTLI 3.

This research evaluates the robustness and adaptability of the power transmission line icing (PTLI) identification scheme by testing it across diverse scenarios with different lighting conditions and backgrounds. The study aims to understand how effectively the method identifies and isolates ice formations in challenging environments by analyzing three sample images, thereby validating its practical applicability in real-world settings. Figure 21 illustrates the tested images under diverse conditions: (a) PTLI 1, (b) PTLI 2, and (c) PTLI 3.

**Figure 22:**
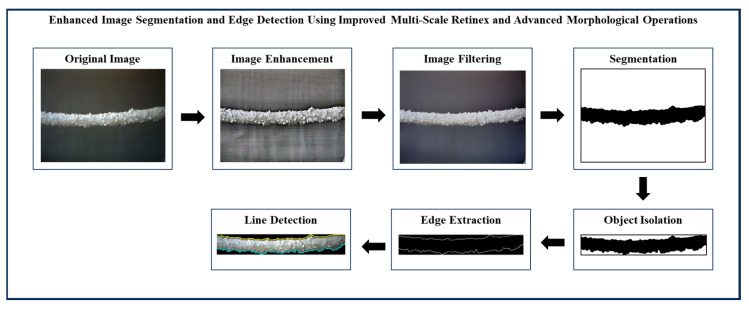
The process of enhanced image segmentation and edge detection using improved multi-scale Retinex and advanced morphological operations applied to PTLI 1.

**Figure 23:**
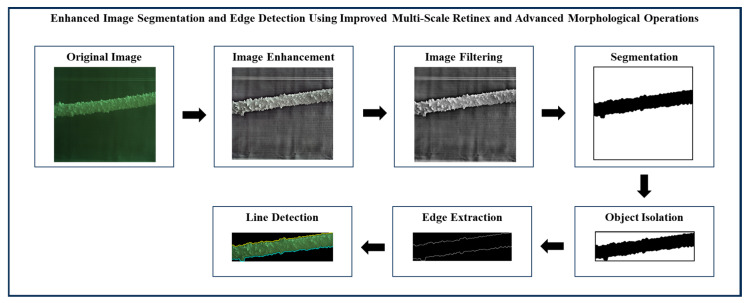
The process of enhanced image segmentation and edge detection using improved multi-scale Retinex and advanced morphological operations applied to PTLI 2.

**Figure 24:**
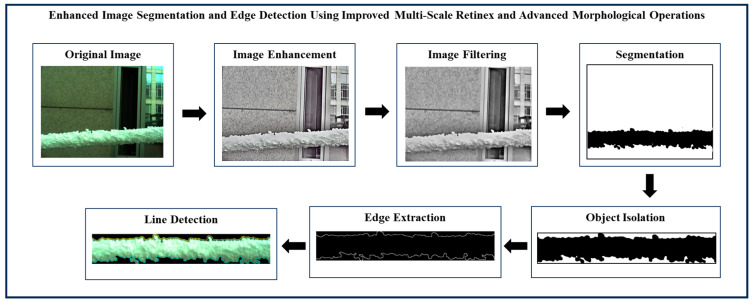
The process of enhanced image segmentation and edge detection using improved multi-scale Retinex and advanced morphological operations applied to PTLI 3.

Figure 22, Figure 23 and Figure 24 illustrate the process of enhanced image segmentation and edge detection using improved multi-scale Retinex and advanced morphological operations. Overall, the figures confirm the methodology’s robustness and versatility in accurately identifying and isolating ice formations under varied conditions, validating its potential for practical use in PTLI identification.

### 3.5. Comparison with Existing Methods

This section evaluates the proposed power transmission line icing (PTLI) identification methodology by comparing its performance with established methods, using ice thickness measurements as the primary benchmark. The comparison is designed to illustrate the improvements and benefits associated with the proposed approach to evaluation, as well as to consider its framework for evaluation, efficiency, precision, and usability when applied to actual scenarios as opposed to other conventional methods.

#### Measurement-Based Ice Thickness Assessment

**Figure 25:**
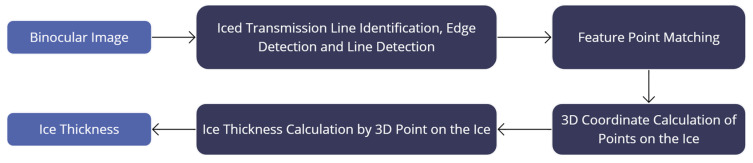
The block diagram of the ice thickness measurement process.

This section evaluates the proposed power transmission line icing (PTLI) identification methodology by focusing on advanced 3D measurement techniques to assess ice thickness. The 3D approach allows for a detailed comparison with existing methods, providing a comprehensive understanding of the proposed method’s accuracy and effectiveness in real-world applications. This analysis aims to validate the method’s precision in measuring ice thickness and identify potential areas for improvement. Figure 25 illustrates the block diagram of the ice thickness measurement process using 3D techniques.

The 3D measurement-based ice thickness assessment involves a multi-step process to accurately measure ice thickness on power transmission lines. It starts with acquiring binocular images, followed by identifying iced transmission lines through edge and line detection. Feature point matching correlates points between images, enabling precise 3D coordinate calculations. These coordinates allow for accurate ice thickness measurement. This comprehensive process ensures reliable monitoring and maintenance of power transmission infrastructure. However, this paper focuses primarily on the iced transmission line identification, edge detection, and line detection processes. The ice thickness measurement is employed not as a primary objective but as a means to evaluate the success of the proposed method. By demonstrating the ability of the proposed methodology to identify and segment ice formations correctly, the paper aims to highlight its effectiveness and reliability.

**Figure 26:**
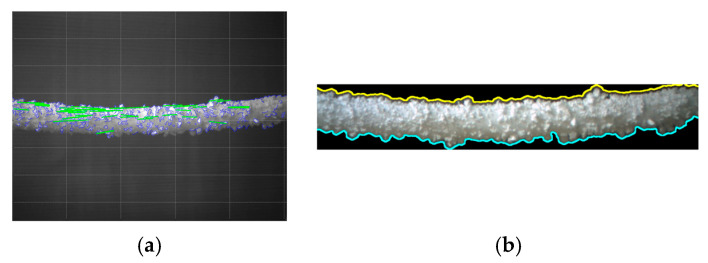
The edge and line detection process: (**a**) The previous method [33,34,37,38]; (**b**) The proposed method.

**Figure 27:**
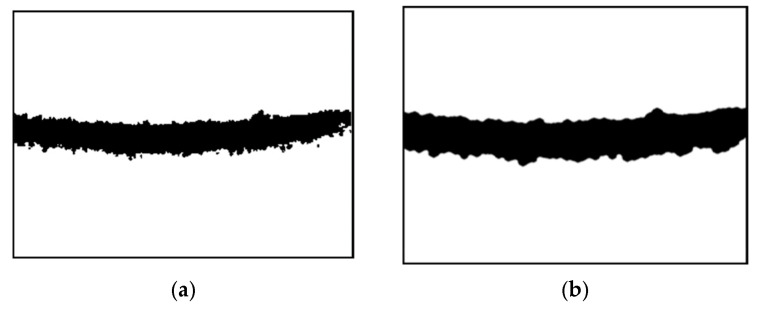
The segmentation process: (**a**) The previous method; (**b**) The proposed method.

Figure 26 compares edge and line detection between a previous method [33,34,37,38] (Figure 26a) and the proposed method (Figure 26b). In the previous method, Canny edge detection (blue) and Hough transform (green) struggle in dimly lit areas, leading to incomplete detection of ice boundaries and impairing ice thickness measurement accuracy. In contrast, the proposed method effectively detects both upper and lower ice boundaries, even under low light conditions, ensuring accurate and comprehensive delineation of the ice formation. This demonstrates the proposed method’s enhanced robustness and effectiveness over traditional approaches. Figure 27 compares the segmentation results between the previous [10] and proposed methods, showing that the proposed method provides clearer and more precise ice boundary delineation.

Table 3 shows the evaluation result using the validation metrics. The proposed method shows significant improvements in accuracy (98.35% vs. 97.1%) and sensitivity (91.63% vs. 86.22%), indicating better detection of true ice formations. Although specificity is slightly lower (99.42% vs. 99.48%), it remains high, and precision is nearly identical (96.03% vs. 96.24%). Overall, the proposed method is more effective and reliable in identifying and measuring ice thickness.

Table 4 presents the results of ice thickness measurements using three methods: the proposed method, Method 1 [33,34,37,38] (previous method), and Method 2 [10] (previous method), compared to manual measurements considered the ground truth. Manual measurement results are measured by a micrometer caliper with an accuracy of 0.05 mm. Method 1 [33,34,37,38] used the Canny transform for edge detection and the Hough transform for line detection. Method 2 [10] utilized an image processing framework for the accurate identification of iced transmission lines, employing multi-level segmentation and mathematical morphology techniques. Previously, Method 1 showed the largest deviation from the manual measurements, indicating lower accuracy. Previously, Method 2 performed better but still showed significant variation. In contrast, the proposed method demonstrates enhanced accuracy and reduced error, offering a more reliable and precise approach to measuring ice thickness.

**Figure 28:**
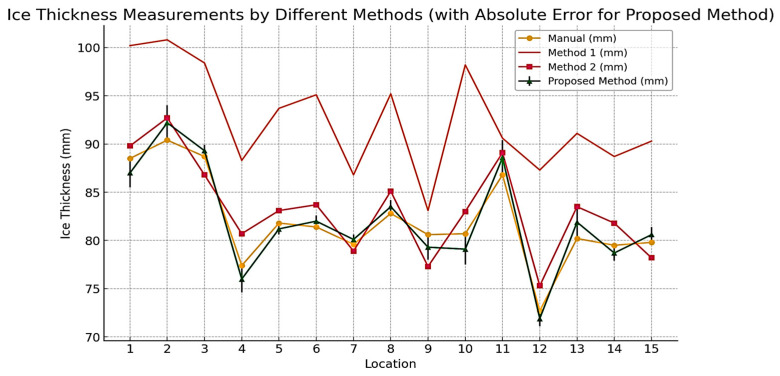
The comparison of the proposed method’s performance with traditional methods under various conditions.

Figure 28 shows the comparison of the proposed method’s performance with traditional methods under various conditions. Figure 28 shows four measurement methods for ice thickness, including manual measurement as a reference, Measurement Method 1, Measurement Method 2, and measurement methods proposed. Measurement Method 1 has the highest difference from the manual measurement method. Method 2 has a smaller difference from manual measurement compared with Method 1. It can also be seen in the graph that Method 2 is positioned below Method 1. Then, the measurement graph on the proposed method looks closer to the manual measurement graph, thus indicating that the proposed method is the most reliable method among the other methods and that the measurement results are almost close to the manual measurement results.

Table 5 highlights the superior performance of the proposed method for ice thickness measurement compared to previous Methods 1 and 2. The proposed method achieves the lowest Root Mean Squared Error (RMSE) at 1.20 mm and the lowest Mean Absolute Error (MAE) at 1.10 mm, indicating high accuracy. It also has a high R-squared (R^2^) value of 0.95, showing strong predictive power and a close fit between predicted and actual measurements. The proposed method demonstrates the least error variability with a standard deviation (SD) of 1.20 mm, emphasizing its consistency and reliability. These metrics collectively highlight the proposed method’s clear advantages in accuracy, consistency, and reliability over the previous methods.

The analysis shows that the proposed method is the most accurate and reliable among the three methods evaluated. It consistently aligns closely with manual measurements, as evidenced by lower RMSE, MAE, and error standard deviation, along with a high R-squared value. In contrast, previous Method 1 has significant inaccuracies and a weaker correlation with manual measurements, while previous Method 2 performs better but still falls short of the proposed method’s accuracy and consistency.

## 4. Conclusions

The new scheme for detecting ice formation on power transmission lines is presented in this paper. The proposed method uses a multistage approach for image processing, such as image enhancement, grayscale conversion, binarization, segmentation, object localization, and edge detection, which has been shown to perform highly under various environmental conditions and is successful in identifying the top and bottom lines of ice load on transmission lines. The presented performance of the proposed method is impressive, with an accuracy of 98.35%, sensitivity of 91.63%, and precision of 96.03% higher than other methods employed. The application of the proposed new scheme indicates that the method used is rather effective, illustrated by performance in the areas of low illumination or background issues. Furthermore, the mean RMSE of 1.20 mm and high R-squared value of 0.95 confirm the accuracy of the ice thickness measurement, and the proposed scheme is useful for monitoring the ice thickness on the power line.

The performance of the proposed method is examined in relation to various lighting and background conditions. It is shown that its performance does not change across scenarios and has high efficiency in detecting and segmenting ice formation on the transmission line. However, there are performance differences noted in some (minor) environments, indicating the need for improvements when used in highly dynamic environments, especially in cases where ice formation is hardly visible due to the background influence. The accuracy metrics obtained from this study have implications for decision-making in managing power transmission line infrastructure. The proposed method improves the likelihood of preventive maintenance by providing nearly accurate and real-time measurement of ice thicknesses on the transmission lines, which in turn makes efficient resource allocation possible and helps reduce the cost effects associated with power outages or maintenance work. The application of binocular vision for ice thickness measurement on transmission lines offers an additional layer of precision that plays a significant role in observing transmission lines in adverse weather.

While the method performed well in controlled environments, there are potential limitations in scaling up this method to real-world-sized systems. For example, the use of high-resolution binocular cameras or the extensive processing that may be required may pose issues regarding cost or scalability at national or international levels. Furthermore, real-time image processing over a large power line network may require further optimization of the computational load. Future work may consider faster analysis and implementation of algorithms or the application of machine learning algorithms for higher scalability requirements but without compromising accuracy.

The conclusion of this study shows the potential of advanced image processing techniques for PTLI monitoring based on 3D measurement but also reveals some directions that require further improvement so that this method can be efficiently scaled up.

## Figures and Tables

**Table 1:** The evaluation metrics.

		Ground Truth	
		PTLI	Not PTLI
The Proposed	PTLI	True Positive (TP)	True Negative (TN)
Method	Not PTLI	False Positive (FP)	False Negative (FN)

**Table 2:** The evaluation results using the sensitivity validation metric.

No	Enhancement (E)	Filter (F)	Multi-Threshold Segmentation (M-S)	Sensitivity	Percentage (%)
1	∨	∨	∨	0.9018	90.18
2	∨	-	∨	0.7798	77.98
3	-	-	∨	0.7369	73.69
4	-	∨	∨	0.6832	68.32
5	∨	∨	-	0.3461	34.61
6	-	∨	-	0.3029	30.29
7	∨	-	-	0.2867	28.67

**Table 3:** The evaluation results using the validation metric.

	Accuracy (%)	Sensitivity (%)	Specificity (%)	Precision (%)
Previous method [10]	97.1	86.22	99.48	96.24
Proposed method	98.35	91.63	99.42	96.03

**Table 4:** The experimental results of ice thickness measurements (mm).

Location	1	2	3	4	5	6	7	8	9	10	11	12	13	14	15
Manual (mm)	88.5	90.4	88.7	77.4	81.8	81.4	79.6	82.8	80.6	80.7	86.8	72.7	80.2	79.5	79.8
Method 1 [33,34,37,38] (mm)	100.2	100.8	98.4	88.3	93.7	95.1	86.8	95.2	83.1	98.2	90.6	87.3	91.1	88.7	90.3
Method 2 [10] (mm)	89.8	92.7	86.8	80.7	83.1	83.7	78.9	85.1	77.3	83	89.1	75.3	83.5	81.8	78.2
Proposed method (mm)	87	92.2	89.3	76	81.2	82	80.1	83.5	79.3	79.1	88.6	71.9	81.9	78.7	80.6
Absolute error	1.5	1.8	0.6	1.4	0.6	0.6	0.5	0.7	1.3	1.6	1.8	0.8	1.7	0.8	0.8

**Table 5:** Key statistical metrics for evaluating.

Metric	Proposed Method	Previous Method 1	Previous Method 2
RMSE	1.20 mm	11.10 mm	2.33 mm
MAE	1.10 mm	10.46 mm	2.21 mm
R^2^	0.95	0.52	0.83
SD	1.20 mm	3.70 mm	1.99 mm

## Data Availability

The original contributions presented in the study are included in the article, further inquiries can be directed to the corresponding author.
